# Paradoxical psoriasis with IL-17 inhibitors

**DOI:** 10.1093/rap/rkae082

**Published:** 2024-07-09

**Authors:** Shikha Singla, Dimitri Luz

**Affiliations:** Department of Rheumatology, Medical College of Wisconsin, Milwaukee, WI, USA; Department of Dermatology, University of Santo Amaro, Sao Paulo, SP, Brazil

Key message

- We highlight the risk of development of paradoxical psoriasis with IL-17 inhibitors.

Dear Editor, The introduction of targeted immune-modulating therapy into the frontline setting has changed the landscape for immune-mediated inflammatory diseases, particularly in the field of dermatology and rheumatology. While substantial progress has been made, the safety profile of currently available therapies is still less than optimal [1]. One of the adverse events that has been a topic of interest is the paradoxical cutaneous reactions with immune-modulating therapies. Paradoxical psoriasis (PsO) has been studied in patients on TNF-α inhibitors. Pugliese *et al*. [2] reported that the incidence rate of paradoxical PsO in patients with inflammatory bowel disease was 5/100 person-years and that smoking is the main risk factor for developing these lesions. In addition to TNF-α, several proinflammatory cytokines such as IL-1β, IL-6, IL-8 and IL-17 are implicated in the pathogenesis of autoimmune diseases that fall under the umbrella of spondyloarthritis [3]. Karamanakos *et al*. [4] conducted a review and linked paradoxical PsO to multiple biologic therapies that inhibit these pro-inflammatory cytokines. In particular, inhibition of the IL-17 axis has been implicated in the development of these reactions. US Food and Drug Administration–approved monoclonal antibodies that inhibit the IL-17 axis have been divided into three categories: anti-IL-17A inhibitors (secukinumab and ixekizumab), anti-IL-17 receptor A inhibitor (brodalumab) and IL-17A/F inhibitor (bimekizumab). IL-17 inhibitors are very effective in controlling disease activity and have been proven to be superior to TNF inhibitors in several head-to-head PsO trials [5]. However, current mechanisms of suppression of the IL-17 axis can lead to complications such as secondary inefficacy due to immunogenicity and the development of IBD. Similar to TNF inhibitors, the cytokine imbalance by inhibition of the IL-17 axis can cause paradoxical PsO [6]. Our objective was to review the published data regarding the risk of development of paradoxical PsO in patients on IL-17 inhibitors.

A literature review was conducted in October 2023 using the PubMed database. Two researchers (D.L. and S.S.) independently searched for terms ‘IL-17 inhibitors’ and ‘paradoxical psoriasis’. Filters were applied to include articles in the English language that involved human subjects. The literature search was not limited by publication date restrictions. To expand the scope of the search, reference lists of the selected articles were reviewed. We excluded review articles (Fig. 1). Data analysis was performed using descriptive statistics. There was no funding source for this study.

**Figure 1.**
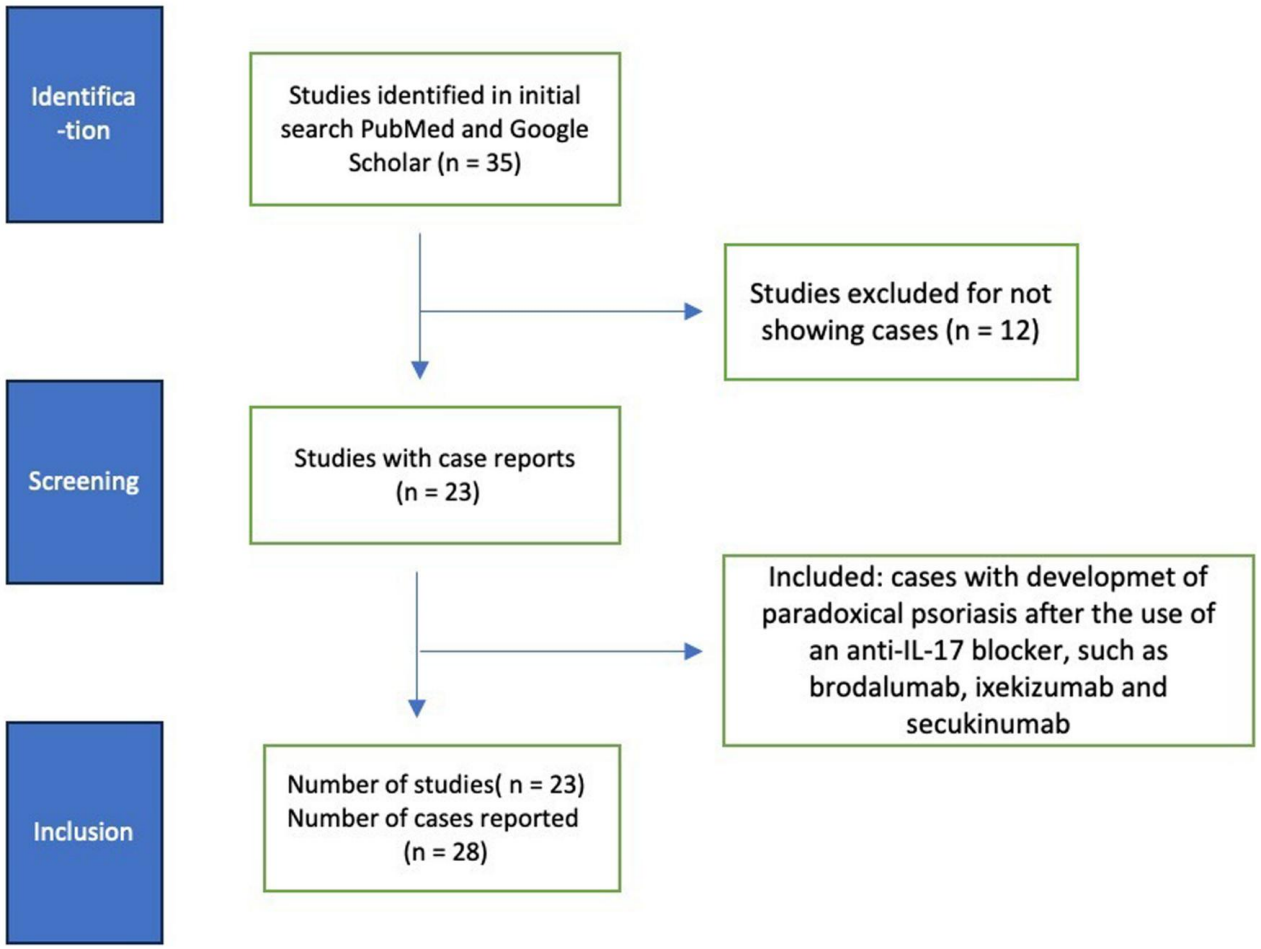
Study design

A total of 35 articles were identified, of which 23 articles met the inclusion criteria encompassing 28 patients with paradoxical PsO due to an anti-IL-17 agent. Among them, secukinumab was prescribed for 58% (*n* = 16), ixekizumab for 21% (*n* = 6) and brodalumab for 21% (*n* = 6) of patients. To date, paradoxical reaction has not been reported with bimekizumab (Supplementary Table S1, available at *Rheumatology Advances in Practice* online).

The majority of patients were females (71%), with the mean age of 51.1 years (range 22–84) and 86% of patients had a prior history of PsO. The most common indication for anti-IL-17 therapy was psoriatic disease (PsO and/or psoriatic arthritis) in 75%, ankylosing spondylitis in 3%, and 2% in both hidradenitis suppurativa and synovitis, acne, pustulosis, hyperostosis, osteitis (SAPHO) syndrome.

Time of onset for paradoxical PsO was <1 month in 14% (*n* = 4), 1 to 6 months in 53% (*n* = 15) and >6 months in 32% (*n* = 9) after the introduction of an IL-17 inhibitor. Seventeen patients presented with palmoplantar pustular lesions. After treatment, 39% of patients had complete resolution and 61% had improvement of their paradoxical condition. Topical glucocorticoids were prescribed for most patients for the treatment of the paradoxical reaction. In addition, four patients received IL-23 inhibitors (two guselkumab and two risankizumab), four received TNF-α inhibitors (three infliximab and one golimumab), three received ciclosporin and three received ustekinumab.

The approach to diagnosis and management of paradoxical PsO with IL-17 inhibitors is varied due to a paucity of data. Our review suggests that female gender, use of secukinumab and psoriatic disease may be potential risk factors for paradoxical PsO. The most common presentation is palmoplantar pustular lesions, which tends to occur 1–6 months after initiating therapy. These findings need to be confirmed by future research.

The development of paradoxical PsO is potentially due to an imbalance in the cytokine pathway, with upregulation of IL-23, IL-22 and TNF-α that occurs when the IL-17 axis is blocked [6]. This mechanism is similar to the paradoxical reactions that occur when the TNF-α pathway is blocked. Suppression of TNF-α induces production of IFN-α, which leads to overexpression of chemokine receptors (e.g. CXCR3) and T cell migration to resident tissues causing an inflammatory reaction [7]. It is important to understand this risk, as there are many new IL-17 inhibitors in the pipeline and early recognition of medication-induced adverse events can prevent harm, distress and long-term disability for patients.

This review is limited by the small sample size and inclusion of case reports. There is the possibility of publication bias, as journals tend to favor reports with positive outcome findings. A meta-analysis could not be performed, as the data were limited, and more placebo-controlled studies are warranted.

In conclusion, we highlight the risk of development of paradoxical PsO in patients on IL-17 inhibitors. Future research, including large placebo-controlled trials are warranted to analyze the significance of this effect. Furthermore, clinicians should carefully assess patients on IL-17 inhibitors for development of paradoxical immune reactions.

## Supplementary Material

rkae082_Supplementary_Data

## Data Availability

The data that support the findings of this study are available from the corresponding author upon request.
