# Supplementary material for: Paradoxical psoriasis with IL-17 inhibitors
Source: Rheumatol Adv Pract. 2024 Jul 9;8(3):rkae082. doi: 10.1093/rap/rkae082 (PMC11257715; doi:10.1093/rap/rkae082)
Supplement: rkae082_Supplementary_Data [file rkae082_supplementary_data.docx]

**Supplementary Table S1: Summary of results**

| **Summary of results (n = 28)** | **Most prevalent (frequency)** |
| --- | --- |
| Sex | Female (71%) |
| Mean age | 51.1 years-old (22-84) |
| Prior history of psoriasis | Yes (86%) |
| Indication of IL-17 inhibitor | Psoriatic disease (PsO and/or PsA) (75%) |
| IL-17 inhibitor | Secukinumab (58%)  Brodalumab (21%)  Ixekizumab (21%) |
| Onset of paradoxical reaction | 1 to 6 months after introduction of drug (53%) |
